# Using spectral and cross-spectral analysis to identify patterns and synchrony in couples’ sexual desire

**DOI:** 10.1371/journal.pone.0205330

**Published:** 2018-10-17

**Authors:** Matthew J. Vowels, Kristen P. Mark, Laura M. Vowels, Nathan D. Wood

**Affiliations:** 1 Department of Electrical and Electronic Engineering, University of Surrey, Guildford, United Kingdom; 2 Department of Music and Media, University of Surrey, Guildford, United Kingdom; 3 Department of Kinesiology and Health Promotion, University of Kentucky, Lexington, Kentucky, United States of America; 4 Department of Psychology, University of Southampton, Southampton, United Kingdom; 5 Department of Family Science, University of Kentucky, Lexington, Kentucky, United States of America; University of Westminster, UNITED KINGDOM

## Abstract

Sexual desire discrepancy is one of the most frequently reported sexual concerns for individuals and couples and has been shown to be negatively associated with sexual and relationship satisfaction. Sexual desire has increasingly been examined as a state-like construct that ebbs and flows, but little is known about whether there are patterns in the fluctuation of sexual desire. Utilizing spectral and cross-spectral analysis, we transformed 30 days of dyadic daily diary data for perceived levels of sexual desire for a non-clinical sample of 133 couples (266 individuals) into the frequency domain to identify shared periodic state fluctuations in sexual desire. Spectral analysis is a technique commonly used in physics and engineering that allows time series data to be analyzed for the presence of regular cycles of fluctuation. Cross-spectral analysis allows for dyadic data to be analyzed for shared rates of fluctuation between partners as well as the degree of (a)synchrony (or phase shift) between these fluctuations. Men and women were found to exhibit fluctuations in sexual desire at various frequencies including rates of once and twice per month, and to have sexual desire that was unlikely to fluctuate over periods of three days or less and therefore exhibited persistence. Similar patterns of fluctuation were exhibited within couples and these patterns were found to be largely synchronous. While instances of desire discrepancy may arise due to differences in rates of sexual desire fluctuation and random fluctuations, such instances may be normal for romantic relationships. The results have important implications for researchers, clinicians, and educators in that they corroborate the supposition that sexual desire ebbs and flows and suggest that it does so with predictable regularity.

## Introduction

Sexual desire is an important component of the human sexual response [1] and sexual desire discrepancy has been identified as one of the most frequently reported sexual issues for couples [2, 3]. Sexual desire can be defined as a goal motivated state of wanting to engage in some form of sexual activity [4, 5]. Previous research has shown that individuals’ levels of sexual desire may ebb and flow [6] leading to instances of sexual desire discrepancy between partners, and subsequently lower sexual and relationship satisfaction [6–8]. While previous research has indicated that hormonal [9–11], relational [12, 13], or social factors [3] may influence levels of sexual desire, to date researchers have not attempted to quantify whether there are regular, cycling, patterns in the fluctuation of sexual desire and assess whether couples exhibit synchrony (i.e. partners’ levels of sexual desire peak and trough at the approximately the same time) or asynchrony (i.e. partners’ peaks and troughs do not consistently coincide) in their fluctuations in sexual desire. The present study utilizes interdisciplinary techniques known as spectral and cross-spectral analysis to investigate the nature of fluctuations in sexual desire. Such techniques may allow researchers and clinicians to understand how sexual desire fluctuates, whether partners’ levels of sexual desire fluctuate at the same frequency, and whether these fluctuations occur in synchrony or asynchrony.

Sexual desire is perhaps surprisingly not relatively well understood [14, 15] especially given its association with sexual and relationship satisfaction [7, 16]. Previous research has also highlighted the positive association between sexual satisfaction and relationship satisfaction [7, 17–20]. Sexual satisfaction may influence the motivation to engage in sexual activity, and research has corroborated the supposition that sexual satisfaction behaves as a mediator between sexual desire and relationship satisfaction [7]. As such, couples experiencing long periods of low sexual desire may see a decline in their sexual satisfaction, which may in turn negatively impact their relationship satisfaction.

Sexual desire discrepancy, where one member of the couple has higher or lower sexual desire relative to their partner, has been shown to be negatively associated with sexual and relationship satisfaction [7, 21]. Further, studies have shown that around one third of women report low sexual desire or desire discrepancy as an issue in their relationship [2, 3]. Recent research has also shown that desire discrepancy may be a natural feature of intimate relationships, and previous research has highlighted how sexual desire may ebb and flow from day-to-day [6, 22] resulting in instances where one partner’s sexual desire is higher than the other’s. For example, in a longitudinal daily diary study of a non-clinical sample of heterosexual couples, Mark’s [22] results showed that both male and female partners had an equal chance of being the lower desiring partner on any given day suggesting that both partners’ sexual desire may fluctuate.

There are several factors that that may influence levels of sexual desire. For example, length of relationship [23], stress [24], childbirth and children [25], and medical illness [26] may have a negative impact on individuals’ levels of sexual desire. Research has also shown that certain medications such as hypertension medications, psychotropic drugs [26], and hormonal contraceptives [27] can reduce sexual desire. However, positive factors also influence sexual desire, such as positive daily relational events [28], and fluctuations in sexual desire may not always be perceived as distressing [29]. Further, research has indicated that a previous day’s desire may predict the following day’s desire [30], indicating persistence or serial correlation.

Previous research has also suggested that there may be regular variation in desire based on hormonal fluctuation in both women and men. For example, a number of studies have indicated that women’s sexual desire may fluctuate according to phases of the menstrual cycle [11, 31–34] highlighting the potential for a gender difference that has biological origins, and may have regular and predictable patterns of change. Female sexual desire has been shown to increase during the follicular phase of the menstrual cycle, peak during the ovulatory phase, and subside at the start of the luteal phase; the probability of engagement in sexual activity is increased during the follicular phase [9, 11].

A 28-day cycle was also identified in men in relationships who were prospective fathers and trying to conceive, but not in men who were single [35]. As such, the 28-day cycle may also be a result of—or reaction to—the menstrual cycle of their female partners [35]. Further, previous research has identified a potential circaseptan (7-day) rhythm in males’ testosterone levels, but the authors were careful to note that sexual activity may precede, rather than succeed, male peaks in hormones [35]; rather than testosterone levels predicting sexual activity, it may have been the sexual activity itself that caused an increase in testosterone.

Sexual desire is often discussed in relation to frequency or regularity. For instance, women’s sexual desire may oscillate according to the menstrual cycle, while men’s may be more irregular and unpredictable, but more frequent and intense [36–40]. Male desire may also be a function of female desire, and vice versa, for mixed-sex couples [35]. Furthermore, even though previous research has suggested that external stressors are not significant in influencing sexual desire, at least not in comparison with internal, relational stressors [3], it still seems reasonable to consider the potential for regular life events to play a part in mediating sexual desire. For example, previous research has shown that mood may vary according to a circaseptan (7-day/weekly) rhythm for men and women, as well as monthly (28 days) in women [41, 42]. As such, there is evidence to suggest that regular, periodic, chronobiological, and chronosocial effects exist, and that there are dyadic interactions.

Sexual desire is an important aspect of romantic relationships that ebbs and flows at a state level, with dyadic implications for couples. While the literature indicates the presence of this state fluctuation in partners’ levels of sexual desire, it is important to further understand whether there are predictable patterns to this fluctuation, and to establish what these patterns look like. Furthermore, if these patterns do exist, it would also be important to understand whether there is dyadic synchrony or asynchrony in these patterns for partners in romantic relationships. Finally, given the potential for serial correlation or persistence in sexual desire, it is also of interest to estimate for how many days sexual desire is likely to be persistent.

Existing data analysis techniques in social sciences do not entirely capture potential periodic, non-linear, fluctuations. This highlights the need to adopt methods from other disciplines that allow us to examine fluctuations in desire within and between couples. The present study utilizes interdisciplinary techniques known as spectral and cross-spectral analysis to investigate the nature of fluctuations in sexual desire. Such techniques may allow researchers and clinicians to understand how sexual desire fluctuates for each individual, whether the rates of fluctuations in levels of sexual desire are shared between partners, and whether these shared rates of fluctuation occur in synchrony or asynchrony. As such, the goals of the study were two-fold. First, to introduce the novel application of spectral analysis with the aim of providing researchers with a different way of understanding the fluctuation of sexual desire over time, and second, the present study aimed to answer the following four specific research questions pertaining to sexual desire:

RQ1: Do participants’ levels of desire fluctuate periodically for men and/or women and if so, at which frequencies?
RQ2: Do participants exhibit persistence in sexual desire?
RQ3: Are there shared rates of sexual desire fluctuation between partners?
RQ4: What is the degree of (a)synchrony between partners’ fluctuations in sexual desire?

## Materials and methods

### Data collection and participants

Two approvals were required for data collection: one from Indiana University IRB (IRB number 1111007329) and the other from University of Kentucky IRB (IRB number 16-0263-P4S-FR60). The current study utilized 30 days of dyadic daily diary data from a non-clinical sample of 133 mixed-sex couples (266 individuals) who were participating in a larger study on sexual desire. Daily diary data are a form of longitudinal data that allow researchers to understand how characteristics and phenomena change daily over time [43]. The participants were asked ‘During the past day, did you feel sexual desire for your partner’ where the options ranged from 1 (‘not at all’) to 5 (‘extremely’). Participants were recruited online, where inclusion criteria required them to be over the age of 18, living with their romantic relationship partner who was of the opposite sex, where their primary relationship partner was also willing to participate in the study.

There were 154 couples who completed the baseline and daily diary portion of the study, although 21 couples completed the baseline but did not continue with the 30-day dyadic daily diary study, indicating a 13.6% dropout rate. Due to the cycling components of daily diary studies [44–46], the total sample consisted of 133 couples (266 individuals) who had fewer than three consecutive missing data points. The remaining missing data points (2.3%) in these 133 couples were imputed using multiple imputation [47–49]. The imputation was based on the linear regression algorithm in SPSS 24, where time was used as the predictor for the imputation of the missing values for the remaining 133 couples in the data set.

The age of participants ranged from 21 to 64 years (men: *M* = 32.27, *SD* = 7.91; women: *M* = 30.91, *SD* = 8.49) where 72.2% of the men were heterosexual and 27.1% were bisexual, and 72.9% of women were heterosexual and 24.8% of women were bisexual. In terms of education level, 49.6% of men had been to college, 20.3% had graduated from college, and 16.5% had been to graduate school. For women, 37.5% had been to college, 25.8% had graduated from college, and 18.2% had been to graduate school. The majority of participants were White (88.0% of men and 90.2% of women). The relationship length ranged from nine months to 31 years (*M* = 6.68, *SD* = 5.99).

### Analysis

The discrete time fast Fourier transform (DFT) algorithm is a computationally efficient algorithm [50] that was utilized to undertake spectral analyses of the groups of 133 men and 133 women separately (RQ1 and RQ2), as well as the dyadic cross power spectral density (cross-spectral) analyses of the 133 couples (RQ3 and RQ4). Both analyses were executed in Matlab R2017b [51]. The equation representing the DFT *X*_*k*_ of time series *x*_*n*_ is shown in Eq (1)

$$\begin{array}{c} X_{k}=\sum_{n=0}^{N-1}W_{N}^{kn}x_{n+1} \end{array}$$ (1)

where *x* is a vector of *n* samples to be transformed and *W*_*N*_ = *e*^−*j*2*π*/*n*^ is the angular frequency, where *j* refers to the square root of −1 [51]. Utilizing the DFT algorithm for dyadic data, the cross power spectral density *P*_*xy*_(*ω*) may then be calculated according to Eq (2) [51–53].

$$\begin{array}{c} P_{xy}\left(\omega \right)=\frac{1}{L}X\left(e^{j\omega }\right)Y^{*}\left(e^{j\omega }\right) \end{array}$$ (2)

where *X*(*e*^*jω*^) is the DFT of time-domain signal *x*, and Y*(*e*^*jω*^) is the complex conjugate of the DFT of the time-domain signal *y*, and *L* is a scaling factor proportional to the length of the signal. This is analogous to a cross-correlation measure, but in the frequency domain.

Both analyses are commonly undertaken in physics and engineering [54] but have been infrequently adopted by social scientists [55, 56]. In the case of spectral analysis, time series data may be transformed into the frequency domain, where regular cycles may be represented as amplitudes or levels of intensity across a range of frequencies [57, 58]. For instance, if an individual’s stress is being measured daily and peaks every Wednesday, the frequency domain representation would contain a peak corresponding with a frequency of once every seven days (i.e. a circaseptan rhythm). In the case of cross-spectral analysis, two time series (e.g. dyadic daily diary data) may be analyzed in order to identify frequencies that are common to both series. In other words, the cross spectral analysis provides an estimation of correlation in the frequency domain. For instance, if a couple had sexual desire that peaks every other weekend, then a cross-spectral analysis of their data would indicate a peak corresponding with a frequency of approximately twice per month. The cross spectral analysis may also yield an estimation of the degree of synchrony or asynchrony between the partners at the identified shared frequencies. The (a)synchrony estimation is quantified in terms of a phase shift between the identified shared frequency components. In this example, where the partners’ levels of desire peak and trough at the same time, the analysis would indicate a discrepancy of approximately zero at the frequency of twice per month.

In order to prepare the data for spectral and cross-spectral analyses, all 266 of the individuals’ time series were standardized so that each individual would have a mean of zero and standard deviation of one. Mean centering was undertaken to reduce any zero frequency offset that contributes bias to low-frequency estimation [44]. Standardization was undertaken in order to mitigate issues associated with averaging across individuals and thereby under-representing those with sexual desire that fluctuates at regular frequencies but at lower amplitudes relative to their standard deviation. Some researchers go so far as to remove any trends from the time series data (e.g. [56]), however, the removal of linear trends may also involve the removal of low-frequency components (e.g. a quarter cycle) and therefore, the removal of linear trends per individual was not undertaken.

For the spectral analyses, the standardized time series were then windowed using the Hann function. Windowing is utilized in order to mitigate spectral estimation bias that occurs when the signal being analyzed starts at a non-zero point, or if the sampling period is not a multiple of the period of the wave being analyzed [59]. Starting at a non-zero point effectively represents truncation of the signal, and reduces the efficacy of the spectral estimation. Windowing is therefore necessary in the analysis of sexual desire as we do not know the period of the sexual desire wave *a priori* to data collection, nor is it possible to begin data collection at a known zero point of sexual desire. Wickramarachi [59] provides a concise review of why the Hann (referred to as ‘Hanning’ in the paper by Wickramarachi [59]) window is suitable in providing good frequency accuracy. This procedure was not undertaken separately for the cross-spectral analyses, because the ‘cpsd’ function in Matlab includes windowing as part of the algorithm.

In order to estimate the average DFTs for the groups of men and women separately, DFTs were taken of each of the 266 individuals’ standardized and windowed time series. The magnitude of these DFTs were averaged across men and women in order to estimate the average spectrum for men and the average spectrum for women. Estimations of spectral significance were undertaken using a variation of bootstrapping, whereby the average spectra were compared 10,000 times against average spectra of 10,000 resorted versions of the men’s data, and 10,000 resorted versions of the women’s data [60, 61]. Each individuals’ standardized time series was resorted 10,000 times and transformed into the frequency domain using spectral analysis. The 2,330,000 resulting spectra were then averaged across 133 men and 133 women separately yielding 10,000 average spectra for men and 10,000 average spectra for women. Finally, the average spectrum for men and the average spectrum for women were compared with the 10,000 average spectra for men and the 10,000 average spectra for women respectively. This process allows for the determination of whether any particular frequency component is statistically significant (i.e. is the spectral component significantly higher in amplitude than that expected in a fully randomized version of the sample time series at least 95% of the time). This process was also adapted by the authors in order to allow for estimations of persistence (i.e. whether the spectral component is significantly lower in amplitude than that expected in a fully randomized version of the sample time series at least 95% of the time).

The cross power spectral densities (CPSDs) of the individual standardized time series of the men and women in each of the 133 couples were then averaged across couples in order to calculate the average cross power spectral density for couples. The cross power spectral density provides an estimate of correlation in the frequency domain. The parameters for the ‘cpsd’ function were as follows: window length—30; window overlap—0; and window type—Hann. The same bootstrapping process described above was then applied with 10,000 iterations across couples instead of individuals in order to establish estimations for statistically significant spectral components and statistically significant persistence between partners in couples. Specifically, each individuals’ standardized data was randomized 10,000 times, yielding 10,000 randomized time series for each partner in each of the 133 couples (2,330,000 randomized time series in total). CPSDs were then computed for each 10,000 randomized versions of each couple’s data. The resulting 1,330,000 CPSDs were then averaged across couples yielding 10,000 CPSDs against which the original, unrandomized, average CPSD could be compared for significance.

The phase between spectral components that are common to both partners may also be derived using the CPSD computation, and describes the amount of lag between partners’ shared spectral components. The phase is given in *π* radians, where 3.14 radians would represent a shared spectral component that is approximately 180 degrees out of phase (i.e. in inverse polarity), and 6.28 radians would represent a shared spectral component that is approximately 360 degrees out of phase, which is also equivalent to 0 degrees in phase. Circular statistics [62] were used to create polar histograms and to calculate the circular mean of the identified significant shared spectral/frequency components for couples in order to allow for an interpretation of the degree of synchrony or asynchrony between partners at these frequencies.

## Results and discussion

Before the time series for each individual participant were standardized, a preliminary analysis indicated that men (*M* = 3.15, *SD* = 1.07) had significantly higher levels of sexual desire, on average, compared to women (*M* = 2.85, *SD* = 1.14), *t*(132) = 4.83, *p* < .001).

### Fluctuation and persistence in individuals

**Fig 1:**
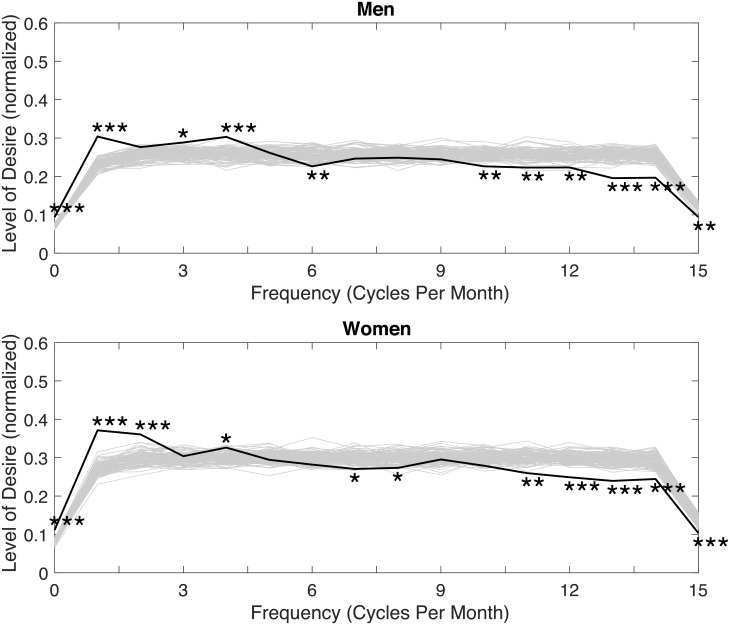
Average DFTs for male and female sexual desire. Includes an illustrative sample (gray lines) of 100 out of the 10,000 spectra of the randomized data. *p < 0.05; **p < 0.01; ***p < 0.001.

The average DFT results for all 133 men and all 133 women are shown in Fig 1. Note that many of the results indicate the presence of a low, indeterminable frequency of ‘zero’ cycles per month. Such a frequency is too low to be meaningful, and may correspond with a trend in the time series. As such, care should be taken not to draw conclusions from instances of this particular frequency. The figure shows that men, on average, fluctuated at frequencies of once (*p* < 0.001), three times (*p* = 0.01), four times (*p* < 0.001), and at a low, indeterminable frequency of zero times per month (*p* < 0.001). Male sexual desire was also found to exhibit statistically significant persistence, and was therefore unlikely to fluctuate at frequencies of six (*p* = 0.01), 10 (*p* = 0.01), 11(*p* = 0.01),12 (*p* = 0.01),13 (*p* < 0.001), and 14 (*p* < 0.001) times per month. Women, on average, fluctuated at rates of once (*p* < 0.001), twice (*p* < 0.001), four times (*p* = 0.04), and at a low, indeterminable frequency of zero times per month (*p* < 0.001). Female sexual desire was also found to exhibit statistically significant persistence, and was unlikely to fluctuate at rates of seven (*p* = 0.03), eight, (*p* = 0.04), 11 (*p* < 0.001), 12 (*p* < 0.001), 13 (*p* < 0.001), 14 (*p* < 0.001), and 15 (*p* < 0.001) times per month.

While a test was not undertaken to establish whether the spectra for men and women were statistically significantly different from each other, the results do indicate similarities. For instance, both men and women, on average, exhibit fluctuation in sexual desire at rates between approximately one and four times per month. Once a month is consistent with previous research suggesting that women’s desire fluctuates during different stages of their menstrual cycle [11, 31–34] and that some men’s desire may fluctuate similarly, potentially due to their partner [35]. However, it is important to interpret these links with caution because we did not examine whether the fluctuation was due specifically to the menstrual cycle, or how many of the female participants were taking hormonal contraceptives that might influence their menstrual cycles. Additionally, previous research has suggested that men’s testosterone levels may fluctuate weekly [35] and both men’s and women’s mood fluctuates based on the circaseptan rhythm [42]. It might, therefore, be plausible to expect that sexual activity and sexual desire also follow such a rhythm. We also identified other regular fluctuations in desire that were significant only for men (three times per month) or women (twice per month) that may be due to other potentially social or biological factors that have not yet been investigated.

Both men and women also exhibit persistence at frequencies between 11 and 15 times per month (where 15 is the highest frequency that could be identified given the sampling frequency of once per day). This means that both men and women may exhibit peaks in their sexual desire anywhere between once every seven to 30 days (approximately), and also are likely to maintain a similar level of sexual desire (and therefore be less likely to fluctuate) over the course of three days (approximately). In other words, consistent with previous research suggesting that the level of desire on one day predicts the level on the next day [30], if sexual desire is high one day then it is likely to remain high for approximately three days, and not peak again for at least another seven days.

### Fluctuation and persistence in couples

**Fig 2:**
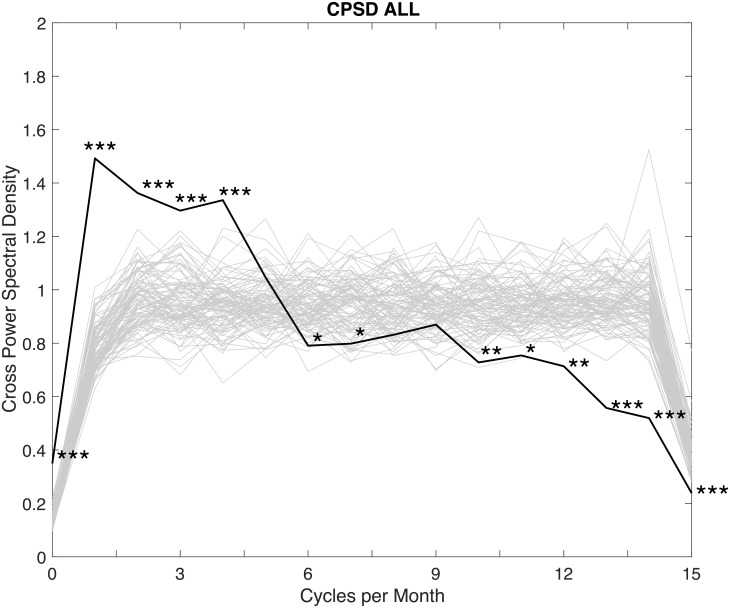
Average cross power spectral density for all 133 couples. Includes and illustrative sample (gray lines) of 100 out of the 10,000 spectra of the randomized data. *p < 0.05; **p < 0.01; ***p < 0.001.

The average cross power spectral density for all 133 couples is presented in Fig 2. It can be seen that couples, on average, exhibit correlation in spectral composition at frequencies of one (*p* < 0.001), two (*p* < 0.001), three (*p* < 0.001), and four (*p* < 0.001) times per month, as well as at a low, indeterminable frequency of zero (*p* < 0.001) times per month. Couples, on average, also exhibit persistence in sexual desire, and, as presented in Fig 2, are unlikely to have sexual desire that fluctuates at rates of six (*p* = 0.03), seven (*p* = 0.05), 10 (*p* = 0.004), 11 (*p* = 0.01), 12 (*p* = 0.004), 13 (*p* < 0.001), 14 (*p* < 0.001), and 15 (*p* < 0.001) times per month. These results indicate that, on average, couples tend to have sexual desire that does not fluctuate over the course of three days (10 times per month), 2.7 days (11 times per month) etc.

**Fig 3:**
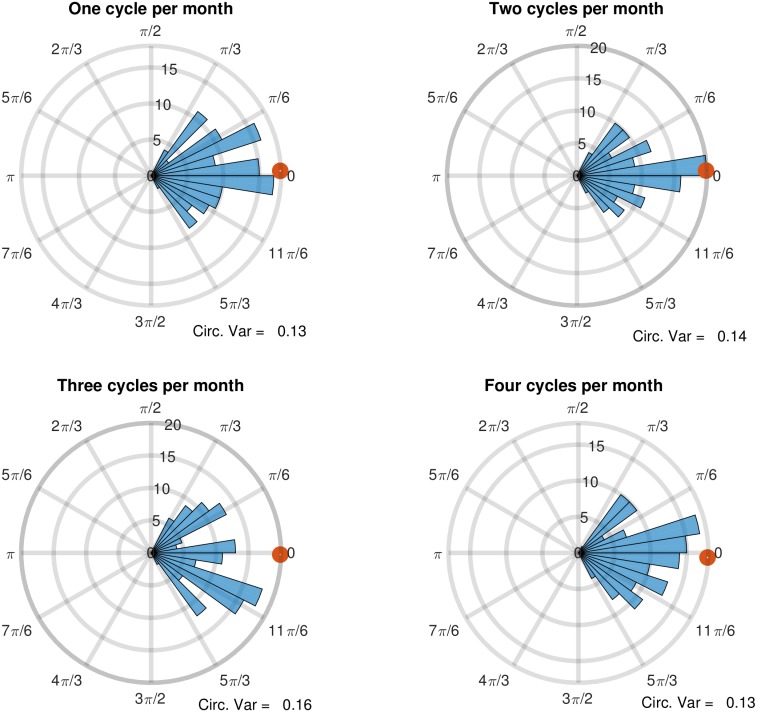
Polar histograms of the four, statistically significant non-zero cross-spectral frequencies. Circular mean phase is indicated by the circular mark on the circumference of the plot (consistently close to 0 degrees.

In order to analyze the phase results and therefore establish an estimation of (a)synchrony between partners, polar histograms were used to visualize the spread of discrepancies, and circular statistics were used to calculate the circular mean and circular variance for each of these statistically significant non-zero cross-spectral frequencies. The polar histograms are shown in Fig 3. It may be seen from the circular average phase (indicated by the circular marks on the circumferences of the polar plots) is approximately zero. This means that, on average, partners in couples have sexual desire that fluctuates in synchrony. The circular variance statistics (which must fall between 0 and 1) provide a measure of the spread of the phase between the partners for each of the four frequencies. While some couples do exhibit phase lead or lag between their shared frequencies of fluctuation, the discrepancies do not exceed approximately ±*π*/3 which is equivalent to ±60 degrees.

In order to interpret these lags with respect to the number of days in the month, a 60-degree phase shift for a frequency with a period of 15 days (i.e. a frequency of twice per month) corresponds with a time discrepancy of 2.5 days (period × phase shift/360). This would suggest that when partner A is at their peak sexual desire, partner B hit their peak 2.5 days before or after partner A, for a couple that cycles twice per month. For a frequency of once per month, which corresponds with a period of 30 days, a 60-degree phase shift corresponds with a time discrepancy of 5 days (30 × 60/360 = 5). Therefore, it appears that couples in this sample are relatively synchronous in their levels of sexual desire when their patterns of fluctuation are similar. Hypothetically, the maximum desire discrepancy would occur with phase discrepancies of 180 degrees, or *π* radians, but couples did not exhibit such a discrepancy.

**Fig 4:**
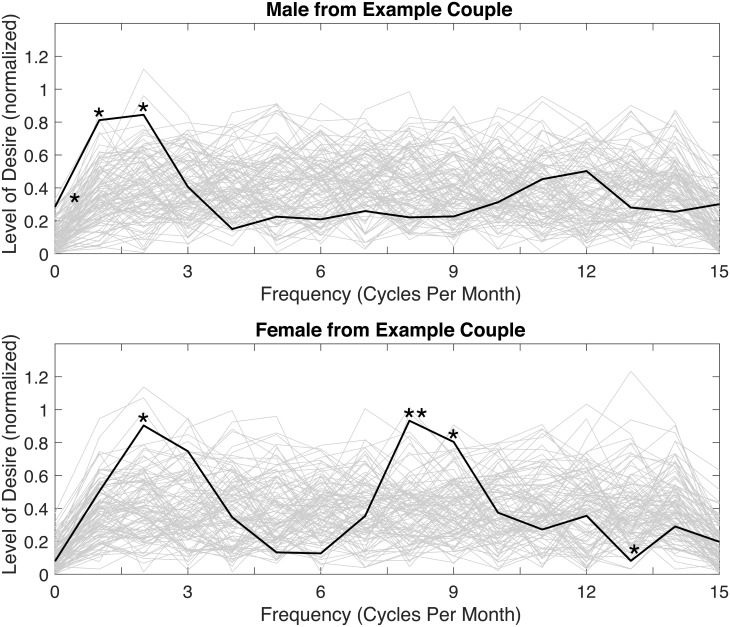
DFTs for two partners in an example couple. Includes an illustrative sample (gray lines) of 100 out of the 10,000 spectra of the randomized data. *p < 0.05; **p < 0.01; ***p < 0.001.

**Fig 5:**
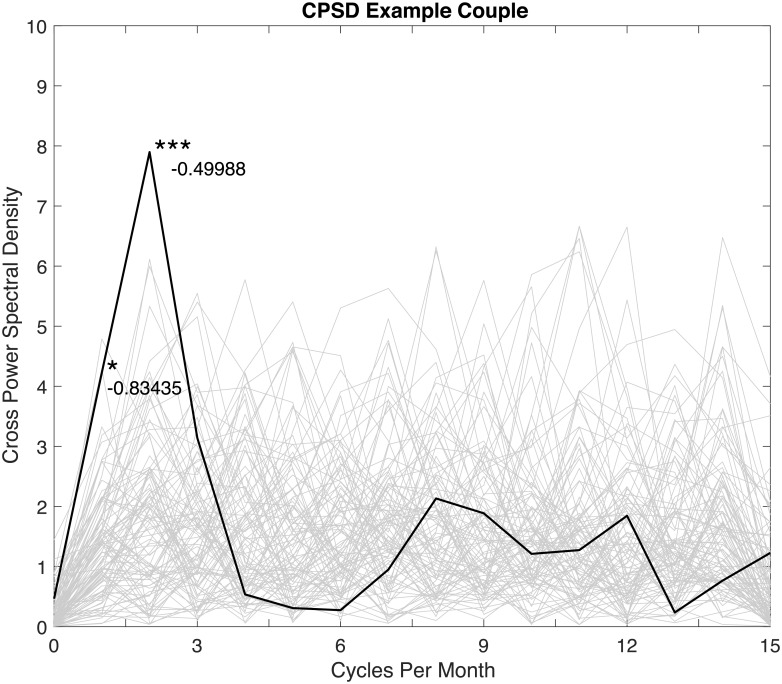
Cross power spectral density for an example couple. Includes an illustrative sample (gray lines) of 100 out of the 10,000 spectra of the randomized data. *p < 0.05; **p < 0.01; ***p < 0.001.

To illustrate how the results might be for a single couple, a DFT of a randomly selected example couple is shown in Fig 4, and their cross power spectral density is shown in Fig 5. Note that the results for this example couple are presented purely for the purposes of demonstration and illustration, and are not intended to be generalized. In this example, it can be seen that the man’s sexual desire fluctuates at frequencies of once (*p* = 0.01) and twice (*p* = 0.02) per month, as well as at some low indeterminate frequency (*p* = 0.03), while the woman’s sexual desire fluctuates at frequencies of two (*p* = 0.01), eight (*p* = 0.01), and nine times per month (*p* = 0.03), and also exhibits persistence and is unlikely to fluctuate at rates of 13 times per month (*p* = 0.03). In other words, while both partners exhibit fluctuation at a frequency of twice per month, the woman exhibits rates of fluctuation that are more frequent and not exhibited by the man. A cross power spectral density analysis of this couple reflects these results. It can be seen in Fig 5 that the couple exhibit statistically significant correlation in the spectral composition of their sexual desire at frequencies of once (*p* = 0.02) and twice (*p* < 0.001) per month but not at eight and nine times per month.

The phase differences of these significant shared frequencies are denoted in Fig 5 as being -0.83 radians (approximately 50 degrees) for the once per month frequency and -0.50 radians (approximately 30 degrees) for the twice per month frequency. At these two frequencies, the phase suggests a lag between the partners of 4.2 days for the once per month frequency and 1.25 days for the twice per month frequency. For small phase lags, a negative phase might be considered to be an instance where the woman’s sexual desire is leading the man’s. However, the sign before the phase is arbitrary and should not be used to infer causality. Given that phase is non-Euclidean (i.e. 360 degrees is equal to 0 degrees) it is impossible to discern between a large lag and a small lead.

**Fig 6:**
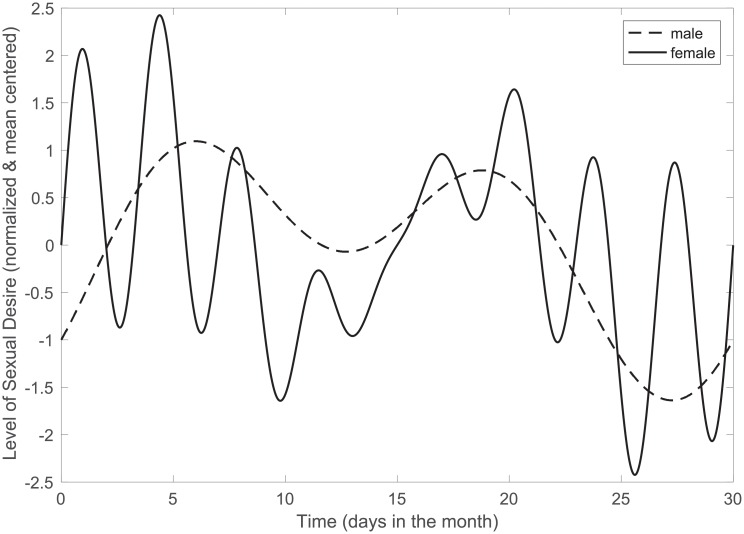
An illustration of the patterns of sexual desire fluctuation for the example couple.

The evolution of the example couple’s sexual desire over the course of 30 days is illustrated in Fig 6. This figure is created by transforming the statistically significant frequency components back into the time domain utilizing the individual spectral estimations for the amplitudes of these shared spectral components. This figure allows one to approximately infer when the couple may exhibit instances of sexual desire discrepancy. For example, approximately eight days into the month, the man exhibits sexual desire that is descending but still high, while the woman exhibits low sexual desire.

### Implications

The results indicate that individuals’ levels of sexual desire exhibit regular, periodic patterns of fluctuation. People also exhibit persistence in sexual desire, meaning that if sexual desire is high (or low) one day, then it is likely to remain high (or low) for approximately the subsequent three days. The results also highlight the potential for partners within a couple to share certain patterns of fluctuation, but also that partners may fluctuate at different frequencies to each other. Such differences may also contribute toward instances of desire discrepancy. However, for frequencies that are shared between partners, these frequencies are likely to be synchronous. As such, desire discrepancy may be more likely to arise as a result of non-shared frequency components, rather than asynchrony between the frequency components.

Our results have several implications for clinicians, educators, and researchers. The results provide further support that sexual desire ebbs and flows, and corroborate the notion that fluctuations in sexual desire (as well as the supposition that any instances of sexual desire discrepancy that occur as a result of these fluctuations) are normal features of the human sexual response. Instead of blaming individuals for instances of low sexual desire, or ‘pathologizing’ desire discrepancy in couples [63], this research helps educators and clinicians understand more about the nature of such discrepancy and sexual desire fluctuation. The results particularly concern clinicians insofar as they should be aware of the nature of sexual desire fluctuation, and the possible relationship between natural fluctuation in sexual desire and the concomitant potential for instances of sexual desire discrepancy between partners. Additionally, researchers should be aware that desire discrepancy exhibits regular fluctuations in both men and women and thus cross-sectional studies of sexual desire may not be able to capture the complexities of sexual desire and thus may be more accurately studied with time series data.

Finally, this study also creates opportunities for further work investigating whether it may be possible to help partners to predict and understand their fluctuations in sexual desire. Indeed, the bootstrapping technique allows for significance estimations for individuals and individual couples (as well as groups of individuals and groups of couples), meaning that clinicians and researchers could conceivably investigate, on a case by case basis, the spectra and cross-spectra of individuals and couples.

### Limitations and further work

There were several strengths in our study including the use of daily time-series dyadic data and a sample of both heterosexual and bisexual individuals and couples. Our study was also the first of its kind to utilize spectral analysis, cross-spectral analysis, and circular statistics in order to identify regular, periodic fluctuations in sexual desire among individuals and synchrony and asynchrony among couples. We also utilized bootstrapping, which allowed us to examine significance of the spectra for individuals, groups, and couples, and without making assumptions about the data (such as the assumption that the data are normally distributed). However, the study was exploratory and the primary purpose was to identify how sexual desire fluctuates over the course of a month rather than specific reasons for the fluctuation. Thus, the study also has several limitations, which are discussed below with suggestions for further work.

There are two main limitations for spectral analysis: First, there is a tradeoff between time domain and frequency domain resolution/accuracy [64–67]. This tradeoff between time domain accuracy and frequency domain accuracy is analogous to the Heisenberg uncertainty principle in that accuracy cannot be achieved simultaneously in both domains [68]. In other words, the longer (and less temporally specific) the time domain sample is, the higher the accuracy in the frequency domain. The shorter (and more temporally specific) the time domain sample is, the less precise the frequency domain representation is. Insofar as the number of time points was limiting, it is generally recommended that the data collection period (30 days in this case) lasts long enough that it contains at least a few cycles of the lowest-frequency cycle of interest [69]. For example, if the menstrual cycle (once per month) is being investigated, then 3-4 months of daily data (or every other day) would allow for more accurate spectral estimations. However, the variance of a spectral estimation increases proportionally to the square of the number of time points [70] and therefore findings of significance may be resultantly overestimated. Further research over a longer period of time is needed to increase the reliability of the lower frequency findings, as well as a more robust estimation of significance and/or a spectral equivalent to effect size measures. Nonetheless, bootstrapping has been demonstrated to be an effective method for testing for significance in the absence of population samples both in cases of limited participant samples size, as well as limited number of time points [60].

Another limitation relates to the sampling frequency. The phenomenon must be sampled at least twice the frequency of the highest frequency of interest [64, 67]. For example, for 30 samples of daily diary data, the highest frequency of a periodic signal that can be identified is one cycle per every two days (because the sampling occurs once a day), which would limit the number of cycles to be analyzed to 15 cycles in total across the 30-day time span. Since the signal is a function of time, and is being represented by functions with regular and stable periodicity, the data must also be sampled at regular intervals. Any component of periodicity that exceeds these bandwidth limitations will result in a phenomenon known as ‘aliasing’ which causes spurious results [71]. Unfortunately, researchers may often have little control over the possibility of aliasing, because, with human participants, the choice of sampling frequency is limited practically. If researchers are concerned with understanding the highest frequency at which sexual desire fluctuates, then the data must be collected at least twice the highest estimated frequency. However, the results from the present study suggests that individuals are likely to exhibit persistence at higher frequencies and their desire is likely to remain high (or low) the following several days. Therefore, once a day may be adequate in assessing individuals’ fluctuations in desire but there may be other phenomena that requires more frequent sampling.

There are also a number of different ‘flavors’ of spectral analysis, which may yield different intuitions. One example is wavelet analysis, which is more robust in analyzing signals that have spectral compositions that change over time (i.e. non-ergodic signals [72, 73]) and may be appropriate for research concerned with human development, for example. Other options include Proper Orthogonal Decomposition (POD) or Spectral Proper Orthogonal Decomposition (SPOD), which are more computationally complex, but may also provide researchers with additional avenues for identifying patterns/commonality within time series data [74–76].

Additionally, the code for the analysis was written by the first author using Matlab 2017b [51]. Undertaking 10,000 randomizations per individual resulted in multiple vectors that were 79.8 million in length. Undertaking cross power spectral density calculations on these vectors and subsequently iterating logical comparisons to estimate significance took over 35 hours to complete on a CPU. However, the code was not optimized for computational efficiency (e.g. replacing iterative loops with vectorized implementations), and there may be opportunity to substantially reduce this execution time.

This study is also fundamentally limited in its capacity to investigate issues pertaining to sexual desire both by the limited diversity of these couples, who were primarily well-educated, White, mixed-sex couples in long-term relationships. A sample comprising same-sex couples and more diverse populations could facilitate interesting comparisons and insights, provided these samples are also of sufficient size to be able to infer meaningful generalizations.

It would be interesting to develop a process whereby the individual DFT results and the individual couple cross-spectral results could be used as predictors for other variables. Further, we recognize that the rates and amplitudes of the fluctuations may relate to other variables such as menstruation, day of the week, trait level sexual desire, and mood. However, there is currently no established combination of spectral analysis with control variables (e.g. day of the week, time of ovulation), and given the novelty of the application it is hoped that such a framework will be developed in the future. For example, in the case of sexual desire, such a framework would allow researchers to investigate whether certain periodicities, or certain amplitudes of certain periodicities, are significant in predicting outcomes such as relationship satisfaction. Further, there is no established effect size measure for spectral analysis in the social sciences, and so future work may attempt to devise a comparative measure. We suggest that the amplitude of the spectral components, relative to the average amplitude of the noise, might be used as a proxy for effect size.

Finally, given the novel application of spectral analysis in the present study, the authors acknowledge that, as with any novel approach, further research may be required to critique, develop, and refine the methodology. It is hoped that this study encourages consideration, exploration, and adoption of non-linear techniques, such as spectral and cross-spectral analysis, for time-series social sciences data.

## Conclusion

The results indicate that individuals’ sexual desire fluctuates at a number of different frequencies including once and four times per month. Individuals were also found to exhibit persistence in sexual desire, in that if sexual desire is high (or low) one day, it is likely to be high (or low) for the next three or four days. Additionally, certain frequency components can be shared between partners in a couple and these shared components were found to be predominantly synchronous, with phase not exceeding *π*/3 radians and an average phase discrepancy of approximately zero. Thus, the results further support the idea that fluctuations in sexual desire and, by implication, sexual desire discrepancy are normal features of intimate relationships. The code developed for this study may also be utilized by clinicians to investigate their individual clients’ patterns in sexual desire.
